# Isolated pulmonary epithelioid hemangioendothelioma: A case report

**DOI:** 10.1097/MD.0000000000040959

**Published:** 2024-12-27

**Authors:** Rong Xuan, Zhengsen Cui, Liuyan Zhao

**Affiliations:** a Department of Endocrinology and Metabolism, Hangzhou Third People’s Hospital, Hangzhou, Zhejiang Province, China; b Department of Geriatric Medicine Department, The First Affiliated Hospital of Zhejiang University, Hangzhou, Zhejiang province, China.

**Keywords:** case report, computed tomography, epithelioid hemangioendothelioma, lung, pleural, surgery

## Abstract

**Rationale:**

Pulmonary epithelioid hemangioendothelioma (P-EHE), initially named “intravascular bronchoalveolar tumor,” is an exceptionally rare malignant tumor with an incidence of <1 in a million. Diagnosis depends primarily on pathological and immunohistochemical findings, and currently, there is no established treatment standard.

**Patient concerns:**

A 50-year-old woman had a physical examination that revealed a lung shadow, followed by a cough and sputum. A chest computed tomography (CT) scan displayed a mass shadow in the upper lobe of the left lung. ^18^F-Fluorodeoxyglucose (^18^F-FDG) positron emission tomography (PET)/CT indicated increased uptake of a clumped shadow.

**Diagnosis:**

The patient underwent a wedge resection of the left upper lung and pleural biopsy, confirming P-EHE.

**Interventions:**

Following P-EHE diagnosis, chemotherapy, targeted therapy, and immunosuppressive therapy are administered.

**Outcomes:**

The patient and his family gave up the treatment because it was not satisfactory. Later, we conducted a phone follow-up and learned that the patient passed away on May 18, 2023.

**Lessons:**

The scarcity of P-EHE complicates its diagnosis and treatment, underscoring the importance of ongoing research to enhance our understanding of this condition.

## 1. Introduction

Epithelioid hemangioendothelioma (EHE) is an exceedingly rare tumor derived from vascular endothelial cells that may manifest in various body parts, including the liver, lungs, soft tissues, and bones.^[1]^ When EHE presents in lung tissue, it is termed pulmonary epithelioid hemangioendothelioma (P-EHE), constituting 19% of all EHE cases and classified as a rare tumor with low to moderate malignancy.^[2]^ This paper reports a case of P-EHE with pleural metastasis and reviews the clinical and pathological characteristics of the disease based on existing literature.

## 2. Case presentation

**Figure 1.**
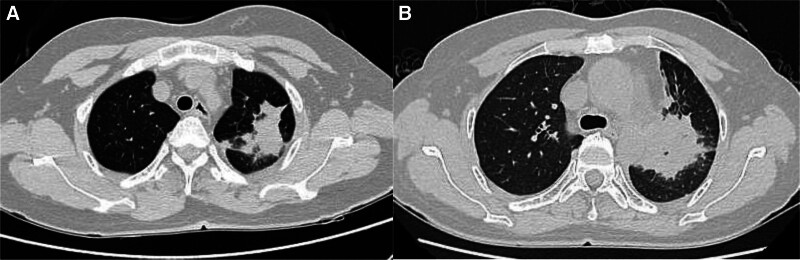
Above are the chest CT images, of which part (A) is the first chest CT image in our hospital, with a lesion measuring approximately 5.1 × 3.6 cm; Part (B) is the second chest CT image, with a lesion measuring approximately 5.9 × 4.6 cm. CT = computed tomography.

**Figure 2.**
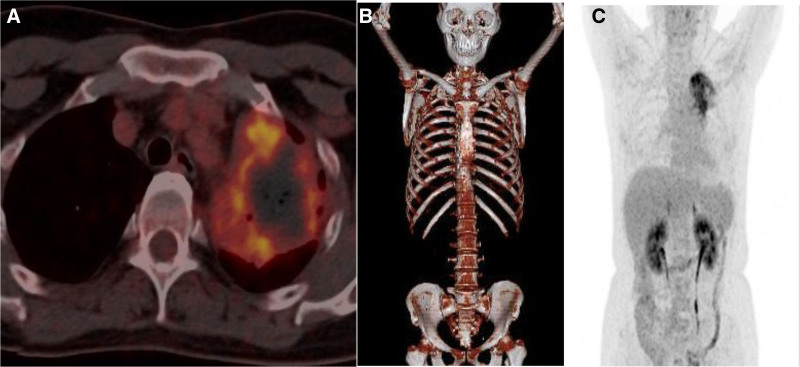
Above are the ^18^F-FDG-PET/CT images, where part (A) shows a high FDG uptake in the upper lobe of the left lung (standardized uptake value maximum of 6.3), and parts (B) and (C) are whole-body images. 18F-FDG PET/CT = ^18^F-fluorodeoxyglucose positron emission tomography/computed tomography, FDG.

**Figure 3.**
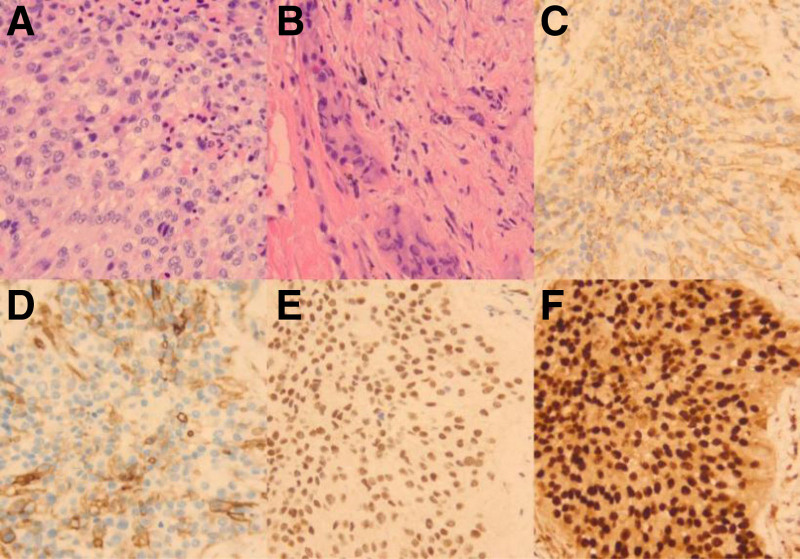
Hematoxylin and eosin stain (×400) of lung and pleural biopsy (A and B). Tumor cells showed strong immunopositivity for CD31 (C), CD34 (D), FLI1 (E) and EGR (F).

**Figure 4.**
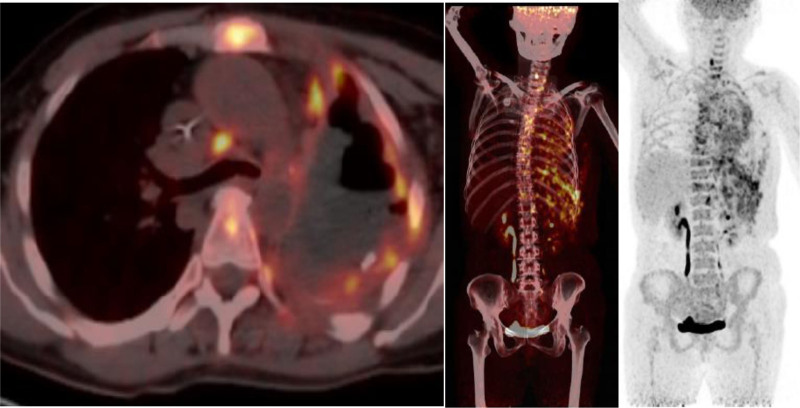
Above is the second 18F-FDG PET/CT images, which show that the lesion has invaded multiple tissues. 18F-FDG PET/CT = ^18^F-fluorodeoxyglucose positron emission tomography/computed tomography.

The patient, a 50-year-old female, was found to have lung shadows during a routine physical examination at an external hospital. She later sought medical attention at our facility due to recurrent coughing and excess phlegm production. She had a history of type 2 diabetes mellitus, denied any history of smoking or tuberculosis, and had no family history of cancer. Laboratory examination revealed hypersensitive C-reactive protein at 30.1 mg/L, a negative tuberculosis infection T-cell test, squamous carcinoma-associated antigen at 0.4 ng/mL, and cytokeratin 19 fragment at 1.1 ng/mL. Our initial chest computed tomography (CT) indicated a mass-like high-density shadow in the upper lobe of the left lung, measuring approximately 5.1 × 3.6 cm with uneven internal density and unclear borders, which was suspected to be inflammatory (Fig. 1A). Despite anti-biotic treatment, the response was poor. A subsequent chest CT showed that the mass in the upper lobe of the left lung had enlarged (5.9 × 4.6 cm), with increased lymph node size in the left hilar and mediastinal areas (Fig. 1B). 18F-fluorodeoxyglucose positron emission tomography/computed tomography (^18^F-FDG PET/CT) examination revealed a nodular, slightly high-density shadow in the upper lobe of the left lung with increased FDG metabolism near the hilum and margin, showing a maximum SUV of approximately 6.3; uneven thickening of the adjacent mediastinal pleura with a mild increase in FDG metabolism, reaching a maximum SUV of about 2.8; and several slightly enlarged lymph nodes in the left pulmonary hilum and mediastinum area 6 with increased FDG metabolism, with a maximum SUV value of approximately 3.1 (Fig. 2). Organizing pneumonia was considered, and treatment with methylprednisolone 40 mg qd was initiated, but the outcome remained unfavorable. To further confirm the diagnosis, thoracoscopic surgery was conducted on September 9, 2022, involving a left upper lung wedge resection, left lower lung pleural biopsy, chest wall pleural biopsy, and lung repair. The surgery revealed the lesion was located in the left upper lung with scattered nodules on the pleural surface. Postoperative pathological examination (Fig. 3) initially suggested an epithelioid vascular endothelial tumor in the upper left lung; heteromorphic cells were also found in the fibrous adipose tissue of pleural nodules, consistent with epithelioid vascular endothelial tumors. Immunohistochemistry was negative for Napsin A, TTF-1, CK (pan), Vimentin, CgA, CD56, Syn, Calretinin, CK7, and EMA, and positive for CD34, INI-1, BRG1, Fli-1, CD31, and ERG. The final diagnosis was P-EHE. Treatment began on September 26, 2022, including chemotherapy (ifosfamide, epirubicin, paclitaxel), targeted therapy (bevacizumab, anlotinib), and immunotherapy (cardonilizumab); however, the treatment outcomes were disappointing. The disease progressed, invading bones, soft tissues, and multiple lymph nodes (Fig. 4), and the patient suffered severe side effects, including Grade IV myelosuppression. Unfortunately, the patient and her family decided to discontinue treatment. A follow-up phone call revealed that the patient had passed away on May 18, 2023; the exact cause of death was not determined.

## 3. Discussion

The average age of onset for P-EHE is around 40 years, and it predominantly affects women; the incidence in women is 2 to 4 times higher than in men.^[3]^ The overall prognosis of P-EHE is favorable, featuring a median survival time of 4.6 years and a 5-year survival rate of approximately 60%.^[4]^ Key prognostic factors include anemia, pleural invasion, lymph node metastasis, and metastasis to distant organs.^[5]^ The clinical symptoms of P-EHE are often nonspecific and may encompass dyspnea, cough, chest pain, hemoptysis, weight loss, and fever. Statistically, nearly 50% of patients are asymptomatic and are diagnosed incidentally during routine physical examinations.^[6]^ P-EHE has the potential to metastasize, affecting pleural and mediastinal lymph nodes, and can spread to the liver, skin, bones, spleen, and central nervous system. In this particular case, the patient was initially identified with a lesion in the left upper lobe during a health screening, later exhibiting symptoms such as cough and sputum production, and eventually presenting with multiple metastases of the lesion.

The most characteristic radiographic feature of P-EHE is the presence of bilateral or unilateral, scattered, small nodules with well-defined edges, generally smaller than 20 mm in diameter, and aligned along the bronchovascular bundles.^[2]^ Isolated lesions in P-EHE are rare, and the largest dimension of these lesions often exceeds 50 mm.^[7]^ In this instance, a chest CT scan identified a single lesion in the upper lobe of the left lung, measuring about 5.1 × 3.6 cm. Compared to CT, ^18^F-FDG PET/CT is more effective in differentiating between benign and malignant tumors and is crucial in locating primary lesions and detecting additional metastases. In this case, the ^18^F-FDG PET/CT revealed a mass-like, slightly hyperdense shadow in the upper lobe of the left lung, increased ^18^F-FDG metabolism in several lymph nodes in the left hilar and mediastinal regions, and mildly increased ^18^F-FDG metabolism in the adjacent mediastinal pleura.

Given that P-EHE lacks specific clinical symptoms, laboratory tests, or imaging characteristics, the definitive diagnosis hinges on pathology and immunohistochemistry findings. Under high-power microscopy, the tumor cells are arranged in strips, nests, or irregular sheets. These cells are epithelioid with abundant eosinophilic, polygonal, or rounded cytoplasm, and some contain small vacuoles. Occasionally, primitive vascular lumens containing single erythrocytes can be observed.^[8]^ Peripheral tumor cells may extend into the surrounding lung tissue along the alveolar septa’s small blood vessels. The alveolar epithelium adjacent to the tumor often exhibits reactive hyperplasia. In rare instances, tumor cells may invade the alveolar or bronchial walls in a dumbbell shape and become infiltrative. Immunohistochemical staining serves as a crucial diagnostic aid for P-EHE, with most tumors expressing vascular antigens such as CD31, CD34, Fli-1, ERG, or Ulex1. CD31 is particularly specific as a vascular tumor marker.^[9,10]^ The immunohistochemistry in this case displayed positive expression of CD31, CD34, ERG, and Fli-1, aiding significantly in establishing the diagnosis.

Since P-EHE is a rare condition, there is no established standard treatment protocol. Generally, if the lesion is solitary and localized, surgical removal is feasible. Notably, both wedge resection and extensive lobectomy offer similar survival outcomes.^[4]^ However, P-EHE typically presents with multiple lesions spanning both lungs, making complete surgical removal impractical. Therefore, chemotherapy and targeted therapy are the recommended treatment options. Radiotherapy is generally ineffective for P-EHE but can be utilized as palliative care for symptomatic relief in other organ metastases, such as bone metastases where it can effectively alleviate pain. Chemotherapy regimens for P-EHE may include adriamycin, cyclophosphamide, paclitaxel, carboplatin, ifosfamide, doxorubicin, vincristine, and actinomycin.^[11]^ As P-EHE originates from blood vessel endothelium, angiogenesis inhibitors such as bevacizumab, apatinib, pazopanib, sorafenib, and sunitinib have been employed in advanced stages.^[12–14]^ Immunosuppressive agents like programmed death 1 (PD-1) inhibitors and programmed cell death-ligand 1 (PD-L1) inhibitors are also being explored for treatment. Due to the malignancy and severe side effects associated with treatment, patients may experience significant distress in advanced stages of the disease, and some treatments may even hasten death. In this case, although the patient initially presented with a single lesion, pleural and lymph node metastases were already evident at the time of surgery. Consequently, chemotherapy, targeted therapy, and immunotherapy were administered postoperatively, yet the patient experienced disease progression and severe adverse drug reactions during treatment.

## 4. Conclusions

In summary, we have detailed a rare, solitary case of P-EHE that was initially mistaken for pneumonia. Our objective is to elevate clinicians’ awareness of P-EHE by sharing our findings, aiming to minimize missed and incorrect diagnoses, thus facilitating timely and effective treatment for patients. Given the absence of a unified and effective treatment standard for this condition, we also anticipate the development of standardized, scientific, and optimized comprehensive treatment strategies for P-EHE through future research efforts.

## Author contributions

**Investigation:** Zhengsen Cui, Liuyan Zhao.

**Methodology:** Zhengsen Cui.

**Resources:** Zhengsen Cui.

**Supervision:** Zhengsen Cui.

**Writing – original draft:** Rong Xuan, Liuyan Zhao.

**Writing – review & editing:** Rong Xuan, Liuyan Zhao.

## References

[1] Blay JY Piperno-Neumann S Watson S; NETSARC/REPPS/RESOS and French Sarcoma Group–Groupe d′Etude des Tumeurs Osseuses (GSF-GETO) networks. Epithelioid hemangio-endothelioma (EHE) in NETSARC: The nationwide series of 267 patients over 12 years. Eur J Cancer. 2023;192:113262. 37625241 10.1016/j.ejca.2023.113262

[2] Huang J Xie S Huang J. Imaging features and deep learning for prediction of pulmonary epithelioid hemangioendothelioma in CT images. J Thorac Dis. 2024;16:935–47. 38505025 10.21037/jtd-23-455 PMC10944745

[3] Chen X Wang Y Che G Shen C. An extremely rare case of pulmonary epithelioid hemangioendothelioma. Thorac Cancer. 2023;14:2519–22. 37488675 10.1111/1759-7714.15051 PMC10447165

[4] Bagan P Hassan M Barthes FLP. Prognostic factors and surgical indications of pulmonary epithelioid hemangioendothelioma: a review of the literature. Ann Thorac Surg. 2006;82:2010–3. 17126100 10.1016/j.athoracsur.2006.06.068

[5] Rosenbaum E Jadeja B Xu B. Prognostic stratification of clinical and molecular epithelioid hemangioendothelioma subsets. Mod Pathol. 2020;33:591–602. 31537895 10.1038/s41379-019-0368-8 PMC7228463

[6] Schattenberg T Kam R Klopp M. Pulmonary epithelioid hemangioendothelioma: report of three cases. Surg Today. 2008;38:844–9. 18751952 10.1007/s00595-007-3712-4

[7] Kitaichi M Nagai S Nishimura K. Pulmonary epithelioid haemangioendothelioma in 21 patients, including three with partial spontaneous regression. Eur Respir J. 1998;12:89–96. 9701420 10.1183/09031936.98.12010089

[8] Gonzalez MF Seth A. Primary pulmonary epithelioid hemangioendothelioma metastatic to the pleura and mediastinal lymph node with a prominent rhabdoid cytomorphology showing CAMTA1::WWTR1 fusion and novel PRDM1 and TNFRSF14 mutations. Cytopathology. 2024;35:654–7. 38943251 10.1111/cyt.13416

[9] Lin H Yuanda C Zhang C. Research progress of pulmonary epithelioid hemangioendothelioma. Zhongguo Fei Ai Za Zhi. 2019;22:470–6. 31315787 10.3779/j.issn.1009-3419.2019.07.10 PMC6712264

[10] Jang YC Hung W-C Su T-C Wu W-P. Primary pulmonary epithelioid hemangioendothelioma. BMJ Case Rep. 2023;16:e254915. 10.1136/bcr-2023-254915 PMC10503352 37709495

[11] Lang XZH. Progress in diagnosis and treatment of pulmonary epithelioid hemangioendothelioma. Cancer Control. 2020;33:262–8.

[12] Zheng Z Wang H Jiang H Chen E Zhang J Xie X. Apatinib for the treatment of pulmonary epithelioid hemangioendothelioma. Medicine (Baltimore). 2017;96:e8507. 29137048 10.1097/MD.0000000000008507 PMC5690741

[13] Semenisty V Naroditsky I Keidar Z Bar-Sela G. Pazopanib for metastatic pulmonary epithelioid hemangioendothelioma—a suitable treatment option: case report and review of anti-angiogenic treatment options. BMC Cancer. 2015;15:402. 25967676 10.1186/s12885-015-1395-6 PMC4437555

[14] Hongfeng NHXYZ. Pulmonary epithelioid hemangioendothelioma: a clinicopathological analysis of four cases. Chin Clin Oncol. 2023;28:836–41.

